# Associations of proteins relevant to MAPK signaling pathway (p38MAPK-1,HIF-1 and HO-1) with coronary lesion characteristics and prognosis of peri-menopausal women

**DOI:** 10.1186/s12944-016-0356-7

**Published:** 2016-11-08

**Authors:** Liqiu Yan, Xufen Cao, Saitian Zeng, Zhe Li, Zheng Lian, Jiawang Wang, Fengfeng Lv, Yunfei Wang, Yanshen Li

**Affiliations:** 1 Department of Cardiology, Cangzhou Central Hospital, Hebei Medical University, No. 16 Xinhua West Road, Cangzhou, Hebei Province 061001 China; 2 Department of Gynecology, Cangzhou Central Hospital, Hebei Medical University, No. 16 Xinhua West Road, Cangzhou, 061001 Hebei Province China

**Keywords:** Perimenopausal CAD, p38MAPK-1, HIF-1, HO-1, Coronary lesion characteristics, Prognosis

## Abstract

**Background:**

The present study was intended to explore whether three proteins within MAPK signaling pathway (i.e. p38MAPK-1, HIF-1 and HO-1) were correlated with peri-menopausal women’s coronary lesion features and prognosis.

**Methods:**

Altogether 1449 peri-menopausal women were divided into non-coronary artery disease (CAD) group (*n* = 860) and CAD group (*n* = 589), including 167 pre-menopausal CAD populations and 422 post-menopausal CAD populations. General information about CAD risk parameters were gathered, including age, family history of CAD or hypertension or diabetes mellitus, bilirubin, cholesterol, triglyceride, high-density lipoprotein cholesterol (HDL-C) and low-density lipoprotein cholesterol (LDL-C) and so on. Coronary angiography results were judged, and CAD score was calculated with application of Genisin scoring method. Besides, detection of MAPK-1 levels was implemented with Strept Avidin-Biotin Complex (SABC) method, while HIF-1 and HO-1 expressions in the serum were determined utilizing ELISA detection kit. Correlations among protein expressions, characteristics of coronary lesions and prognosis of CAD populations were finally evaluated.

**Results:**

Hypertension, hyperlipoidemia, diabetes and smoking history were more prevalent among postmenopausal CAD women than premenopausal CAD women (*P* < 0.05). Furthermore, postmenopausal women seemed to be significantly associated with multiple (i.e. double and triple) vessel lesions and severe lesion types (type B and C), when compared with premenopausal CAD group (*P* < 0.05). Similarly, remarkably elevated expressions of p38MAPK-1, HIF-1 and HO-1 were found within postmenopausal CAD populations in comparison to premenopausal ones (*P* < 0.05). The internal CysC, hs-CRP, TG and LDL-C concentrations all accorded with the following tendency: postmenopausal CAD women > premenopausal CAD women > non-CAD women. Moreover, p38MAPK-1, HIF-1 and HO-1 expressions were up-regulated with increasing number of vessel lesions and severity of coronary lesions among peri-menopausal women. Besides, among both pre-menopausal and post-menopausal CAD groups, positive correlations could be observed between MAPK-1 and TG (*r*_s_ = 0.271; *r*_s_ = 0.476), between HIF-1α and LDL-C (*r*_s_ = 0.077; *r*_s_ = 0.470), as well as between HO-1 and CysC (*r*_s_ = 0.492; *r*_s_ = 0.190) or hs-CRP (*r*_s_ = 0.569; *r*_s_ = 0.542) (all *P* < 0.05). MAPK-1, HIF-1α and HO-1 were also, respectively, positively correlated with CysC (*r*_s_ = 0.415), hs-CRP (*r*_s_ = 0.137), and TG (*r*_s_ = 0.142), regarding post-menopausal CAD women (all *P* < 0.05). Finally, only SBP and TG were regarded as independent risk factors for CAD prognosis (i.e. high Genisin score) among premenopausal women (OR = 1.02, 95%CI: 1.01–1.18, *P* = 0.043; OR = 1.82, 95%CI: 1.01–3.33, *P* = 0.047).

**Conclusions:**

Expressions of p38MAPK-1, HIF-1 and HO-1 could serve as predictive roles for coronary lesions among peri-menopausal women.

## Background

Cardiovascular disorders, especially coronary artery disease (CAD), always stand high in their causal mortality and morbidity [1]. It has been stressed that CAD was more prevalently found in men than in women for that estrogen can, to some extent, safeguard women from risk of CAD [2]. Nonetheless, emerging documentaries suggested that women who were below 50 years old appeared to be associated with 2 folds of mortality rate when compared with men of matched ages [3]. The potential account for this distinct contrast could be hypothesized as hypoestrogenaemia, apart from certain well-established classical risk elements, including smoking history, hypertension, hyperlipidemia, diabetes and so on [4]. Epidemiological data has also confirmed a remarkable rise of CAD prevalence among postmenopausal populations, despite a lower susceptibility to CAD among premenopausal women than that within men [5]. However, due to the under-estimation of CAD among women, systematic evaluations targeting perimenopausal women and relevant underlying mechanisms still remained lacking [6].

Diverse proteins have been speculated as reliable serum biomarkers for susceptibility to CAD, such as adipocyte fatty acid-binding protein (A-FABP), retinol-binding protein-4 (RBP4), hypoxia inducible factor-1 (HIF-1), heme oxygenase-1 (HO-1) and so on [7–10]. Notably, HIF-1 was subject to regulation of estrogen via correlated Akt and p38-mitogen activated protein kinase (p38MAPK) pathways, implying that HIF-1 might emerge as the featured biomarker between premenopausal and postmenopausal populations [11]. In addition, since HIF-1/HO-1 signaling pathway was involved with the molecular mechanism that contributed to development of myocardial ischemia, HO-1 was also incorporated as a candidate protein that could be modified by estrogen [12, 13]. Besides, p38MAPK appeared to be commonly investigated in the area of myocardial inflammation, rather than CAD, yet it functioned as the upstream molecule of HIF-1 [14]. As myocardial inflammation was highly correlated with CAD [15], p38MAPK could also count much in differentiating CAD from non-CAD, even in marking the discrepancies between premenopausal and postmenopausal CAD.

Above all, multiple factors related with CAD for women were distinct from that for men, regardless of clinical manifestations, pathogenesis, diagnosis, drug metabolism and prevention strategies. The present study was intended to explore characteristic proteins that could be tightly linked with perimenopausal CAD risk and coronary artery lesions, providing solid evidence for further exploration of treatment targets for CAD.

## Methods

### Subjects

Altogether 1449 peri-menopausal women suspected with CAD were checked with coronary angiography in Cangzhou central hospital from January 2007 to June 2010. For retrospective analysis, the subjects were divided into non-CAD group (*n* = 860) and CAD group (*n* = 589), which was then sub-grouped into pre-menopausal CAD group (*n* = 167) and post-menopausal CAD group (*n* = 422). The baseline characteristics of the participants were recorded, including history of diabetes, hypertension, hyperlipidemia and myocardial infarction. We also gathered information relevant to medications and treatment strategies managed for the subjects, as well as the results of coronary angiography. The participants were included into the CAD group when at least one of their vascular lumens within left main (LM), left anterior descending (LAD), left circumflex artery (LCX) and right coronary artery (RCA) showed a diameter stenosis of more than 50 %. Besides, patients satisfying the following criterion would be excluded from our study: (1) they have incomplete medical records; (2) they simultaneously suffered from other myocardial disorders, such as dilated cardiomyopathy and rheumatic heart disease; (3) they concurrently have other severe diseases related with liver, kidney, blood, tumor, endocrine, immunity system and so on; (4) they suffered from amenorrhoea due to diverse causes or underwent estrogen replacement treatment (ERT); (5) they were diagnosed as psychosis; (6) they had history of operation or trauma within 1 month. All participants have signed informed consents, and the present study was approved by the ethics committee of Cangzhou central hospital, Hebei province.

### Detection of biochemical indicators

Early next morning after hospitalization, fasting venous food were drawn from patients, and fully automatic dry biochemical analyzer (model: OLYMPUS-AU-5400) was applied to detect certain biochemical indicators, including levels of bilirubin, cholesterol, triglyceride, high-density lipoprotein cholesterol (HDL-C) and low-density lipoprotein cholesterol (LDL-C). Besides, blood routine indicators, which mainly included leucocyte count, neutrocyte proportion, red blood cell count and haemoglobin level, were determined by blood analyzer (model: LH 750), and fibrinogen levels were checked with coagulation analyzer (model: ACL TOP).

### Coronary arteriongraphy

Right radial artery or femoral artery was pierced with Seldinger method, and they were projected with Judkin method in multiple perspectives and multi-positions. Left coronary artery was projected with ≥ 4 positions, while right coronary artery was projected with ≥ 2 positions. The arteriography results were reviewed and judged by experienced cardiologists and interventional physicians. Based on the count of stenosed coronary vessel, CAD lesions were categorized into single-vessel lesions, double-vessel lesions and triple-vessel lesions. Diagonal branch lesions were regarded as LAD, while blunt circular lesions were deemed as LCX. Posterior descending lesions and left ventricular lesions were incorporated into RCA, and LM was calculated as a single lesion. Moreover, considering the complexity of the CAD lesions, they were classified as type A, type B and type C in accordance with the guidelines of American College of Cardiography (ACC)/American Heart Association (AHA).

### Genisin scoring

The degree of coronary artery stenosis was quantitatively assessed by Genisin scoring: (1) 0 score when proportion of stenosis = 0; (2) 1 score when proportion of stenosis ≤ 25 %; (3) 2 scores when proportion of stenosis achieved 26 % ~ 50 %; (3) 4 scores when proportion of stenosis achieved 51 % ~ 75 %; (4) 8 scores when proportion of stenosis achieved 76 % ~ 90 %; (5) 16 scores when proportion of stenosis achieved 91 % ~ 99 %; (6) 32 scores when proportion of stenosis achieved 100 %. Different stenosis segments of coronary arteries were based on Genisin scores multiplied by corresponding coefficients: (1) x 5 for LM; (2) x 2.5 for proximal ends of LCX and LAD; (3) x 1.5 for medium-ends of LCX and LAD; (4) x 1 for far-ends of RCA/LAD/LCX, first diagonal branch, second diagonal branch, posterior descending branch, posterior collateral branch and obtuse marginal branch. The stenosis score of coronary arteries for each patient was the sum score of all branches. Finally, five groups were drawn based on Genisin scores: (1) group 1 (0 < sum ≤ 10); (2) group 2 (10 < sum ≤ 20); (3) group 3 (20 < sum ≤ 34); (4) group 4 (34 < sum ≤ 64); (5) group 5 (sum > 64).

### Definition of risk indicators

The participants would be diagnosed with hypertension if their systolic pressure was ≥ 140 mmHg and/or diastolic pressure was ≥ 90 mmHg for three times in different days without taking anti-hypertensive drugs. Diabetes was defined as the station that blood sugar level was ≥ 11.1 mmol/L at any time, or fasting blood-glucose (FDG) was ≥ 7.0 mmol/L, or blood sugar level remained ≥ 11.1 mmol/L 2 h after loading [16]. The diagnosis of hypertension accorded with the criteria that the sitting blood pressure of participants were ≥ 130/85 mmHg. Smoking history was confirmed if subjects had habits of regular smoking previously, or they smoked ≥ 1 cigarette/day for ≥ 1 year.

### Detection of MAPK-1 levels with Strept Avidin-Biotin Complex (SABC) method

Paraffin sections of samples were routinely placed in xylene for transparent dewaxing and gradient alcohol for hydration. After rinsing with distilled water for 3 times (5 min for each time), the sections were immersed in 3 % H2O2 at room temperature for 10 min to inactivate endogenous enzymes. Then the sections were immersed in 0.01 M citrate buffer solution (pH6.0) and simultaneously heated to boiling. When the boiled solution was cooled, it was cleansed with phosphate buffer saline (PBS) (pH7.2-7.6) for 1–2 times. The bovine serum albumin (BSA) sealing fluid was then dipped at room temperature for 20 min. The primary antibody for p-MAPK (Stancruz corporation, USA) was firstly dipped to incubate tissues at 37 °C for two hours, and then biotin goat-anti-mouse secondary antibody was dipped at 37 °C for 20 min. Subsequently, the reagent of SABC was dipped at the temperature ranging from 20 to 37 °C for 20 min. Finally, DAB color developing reagent kit was applied to color the tissues, and hematoxylin was employed to redye the tissues. The sections were observed under the microscope and corresponding integrated optical density (IOD) was processed with application of Image pro plus software.

### Evaluations of HIF-1 and HO-1 levels

The concentrations of HIF-1 and HO-1 in the serum were detected with usage of ELISA detection kit (Shanghai langdun biotech corporation, China). The sensitivities of HIF-1 and HO-1 were 2 ng/L and 50 ng/L, respectively. The experimental procedures were strictly in accordance with the instructions of the kit. Specifically, HIF-1 and HO-1 were added to the enzyme labeled pores that were pre-packaged with antibodies of HIF-1 and HO-1, and were then incubated. After cleansing, HIF-1 and HO-1 antibodies that were marked with horseradish peroxidase (HRP) were added, and then substrates of A and B were supplemented to generate blue, which was finally converted to yellow under the influence of acid. The depth of colour was positively correlated with concentrations of HIF-1 and HO-1 in samples.

### Statistics

All the statistical analyses were conducted on the basis of SPSS 18.0 software. Measurement data (mean ± SD) were compared with student's *t* test, while enumeration data in the form of percentages (%) were compared with chi-square test. Data of each group were examined to confirm whether they conformed to normal distribution and homogeneity of variance. Multi-group comparisons were conducted with application of analysis of variance (ANOVA). Correlation analysis was performed with Pearson analysis. The regression model was established to explore independent risk factors for premenopausal and postmenopausal CAD. It was considered statistically significant when *P* values were found to be less than 0.05.

## Result

### Baseline characteristics of subjects

Participants in non-CAD group, pre-menopausal CAD group and post-menopausal CAD group were well matched in terms of BMI and age without statistical significance (*P* > 0.05) (Table 1). The occurrence of either CAD or menopause seemed to be associated with distinct incidences of hypertension, hyperlipoidemia, diabetes (*P* < 0.05). Family history and smoking history might also render development of CAD to be significantly distinct (*P* < 0.05), yet the prevalence of obesity was similar in this population (*P* > 0.05).

**Table 1:** Comparison of baseline clinical characteristics among groups of non-CAD, premenopausal CAD and postmenopausal CAD

Indicators	Non-CAD group	Premenopausal-CAD group	Postmenopausal-CAD group	F/*X* ^2^	*P* value
Size	860	167	422		
BMI (kg/m^2^)	24.1 ± 2.4	24.3 ± 3.1	24.0 ± 2.1	1.0	0.36
Age (years old)	50.7 ± 2.7	50.8 ± 2.9	50.7 ± 1.2	2.4	0.09
Hypertension					
yes	291 (33.8 %)	70 (41.9 %)	229 (54.3 %)	49.1	<0.05
no	569 (66.2 %)	97 (58.1 %)	193 (45.7 %)		
Hyperlipoidemia					
yes	204 (23.7 %)	37 (22.2 %)	141 (33.4 %)	15.4	<0.05
no	656 (76.3 %)	130 (77.8 %)	281 (66.6 %)		
Diabetes					
yes	108 (12.6 %)	29 (17.4 %)	124 (29.4 %)	54.3	<0.05
no	752 (87.4 %)	138 (82.6 %)	298 (70.6 %)		
Family history					
yes	140 (16.3 %)	64 (38.3 %)	75 (17.8 %)	44.6	<0.05
no	720 (83.7 %)	103 (61.7 %)	347 (82.2 %)		
Smoking history					
yes	151 (17.6 %)	55 (32.9 %)	96 (22.7 %)	21.4	<0.05
no	709 (82.4 %)	112 (67.1 %)	326 (77.3 %)		
Obesity					
yes	192 (22.3 %)	43 (25.7)	116 (27.5%)	4.4	0.11
no	668 (77.7 %)	124 (74.3 %)	306 (72.5 %)		

### Comparison of coronary angiography indicators

Whether menopause occurred or not might not be associated with the location of lesion vessels, since that no significant difference was observed between the two groups regarding lesion vessels within left main coronary artery, anterior descending artery, circumflex coronary artery and right coronary artery (*P* > 0.05) (Table 2). However, post-menopause could be correlated with larger proportions of double/triple vessel lesions and higher degrees of lesion type (type A and type B) than pre-menopause (*P* < 0.05).

**Table 2:** Comparison of coronary angiography indicators between groups of premenopausal CAD and postmenopausal CAD

Indicators	Premenopausal-CAD group	Postmenopausal-CAD group	*X* ^2^	*P* value
Lesion vessels				
Left main coronary artery	6 (3.6 %)	18 (4.3 %)	1.1	0.78
Anterior descending artery	97 (58.1 %)	279 (66.1 %)		
Circumflex coronary artery	92 (55.1 %)	269 (63.7 %)		
Right coronary artery	72 (43.1 %)	246 (58.3 %)		
Number of stenosed coronary vessel				
Single vessel lesion	103 (61.7 %)	175 (41.5 %)	19.7	<0.05
Double vessel lesion	28 (16.8 %)	104 (24.6 %)		
Triple vessel lesion	36 (21.5 %)	143 (33.9 %)		
Lesion type				
Type A	59 (35.3 %)	53 (12.6 %)	40.6	<0.05
Type B	71 (42.5 %)	230 (54.5 %)		
Type C	37 (22.2 %)	139 (32.9 %)		

### Comparison of protein expressions relevant to MAPK signaling pathway

Expressions of MAPK-1, HIF-1α and HO-1 in the pre-menopausal group were found to over-number those in the non-CAD group (*P* < 0.05), and the three proteins in the post-menopausal group expressed even higher than those in the pre-menopausal group (*P* < 0.05) (Table 3).

**Table 3:** Comparison of protein expressions (MAPK-1, HIF-1α and HO-1) among groups of non-CAD, premenopausal CAD and postmenopausal CAD

Proteins	Non-CAD group	Premenopausal CAD group	Postmenopausal CAD group
MAPK-1^a^	3.5 ± 0.1	4.5 ± 0.1^b^	4.6 ± 0.1^b,c^
HIF-1α (ng/L)	9.4 ± 2.5	20.7 ± 6.1^b^	24.4 ± 7.6^b,c^
HO-1 (ng/L)	136.6 ± 52.8	382.1 ± 135.4^b^	413.7 ± 112.9^b,c^

^a^integrated optical density (IOD) values

^b^compared with non-CAD group

^c^compared with pre-menopausal CAD group

**Fig. 1:**
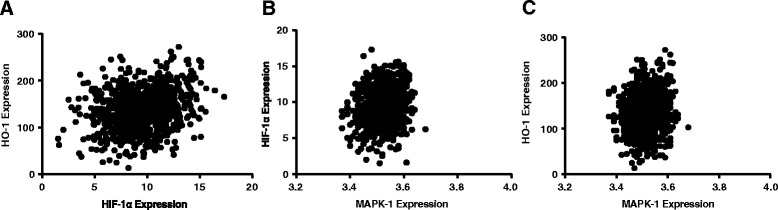
Correlations among expressions of p38MAPK-1, HIF-1α and HO-1 in non-CAD group

**Fig. 2:**
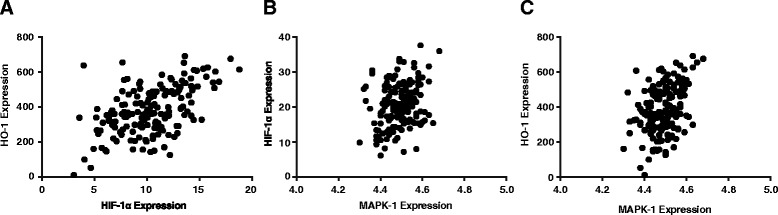
Correlations among expressions of p38MAPK-1, HIF-1α and HO-1 in pre-menopausal CAD group

**Fig. 3:**
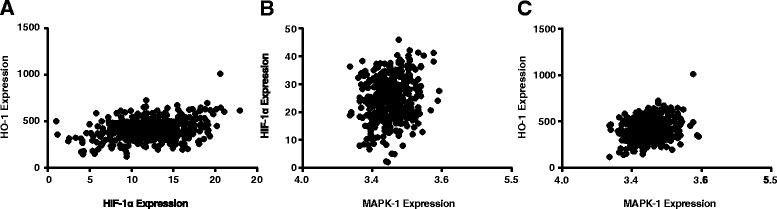
Correlations among expressions of p38MAPK-1, HIF-1α and HO-1 in post-menopausal CAD group

Additionally, significant correlations were found in the non-CAD group, respectively, between HO-1 and HIF-1 (*r*_s_ = 0.25, *P* < 0.05), p38MAPK and HIF-1 (*r*_s_ = 0.20, *P* < 0.05), p38MAPK and HO-1 (*r*_s_ = 0.12, *P* < 0.05) (Fig. 1). Pre-menopausal group and post-menopausal group also exhibited the similar tendencies of the three relations as non-CAD group, which were specifically displayed as: HO-1 and HIF-1(*r*_s_ = 0.52, *P* < 0.05; *r*_s_ = 0.34, *P* < 0.05), p38MAPK and HIF-1 (*r*_s_ = 0.28, *P* < 0.05; *r*_s_ = 0.20, *P* < 0.05), p38MAPK and HO-1 (*r*_s_ = 0.38, *P* < 0.05; *r*_s_ = 0.23, *P* < 0.05) (Figs. 2 and 3).

### Comparison of CAD-related indicators

Among the thirteen indicators, the measured values of around five parameters (i.e. SBP, CysC, hs-CRP, TG and LDL-C) all conformed to the following tendency with statistical significance: post-menopausal group > pre-menopausal group > non-CAD group (*P* < 0.05) (Table 4). Nonetheless, the remaining indicators were approximate in their detection values among groups of non-CAD, pre-menopausal CAD and post-menopausal CAD (*P* > 0.05).

**Table 4:** Comparison of CAD-related indicators among groups of non-CAD, premenopausal CAD and postmenopausal CAD

Indicators	Non-CAD group	Premenopausal CAD group	Postmenopausal CAD group
SBP (mm Hg)	123.0 ± 14.7	134.9 ± 16.3^a^	140.5 ± 18.1^a,b^
DBP (mm Hg)	80.5 ± 9.2	83.8 ± 10.5	82.6 ± 9.7
Heart rate (/min)	86 ± 10	88 ± 17	88 ± 14
FBG (mmol/L)	7.6 ± 1.5	9.3 ± 0.8	10.4 ± 1.3
2 h PG (mmol/L)	12.3 ± 1.9	15.2 ± 2.3	15.9 ± 2.1
BUN (mmol/L)	6.9 ± 1.6	7.7 ± 2.7	8.2 ± 3.5
Cr (μmol/L)	76.7 ± 16.5	84.1 ± 24.1	88.8 ± 21.9
CysC (mg/dL)	1.1 ± 0.2	1.4 ± 0.3^a^	1.8 ± 0.2^a,b^
hs-CRP (mg/L)	0.7 ± 0.2	12.8 ± 8.3^a^	16.5 ± 7.9^a,b^
TC (mmol/L)	4.4 ± 0.7	4.7 ± 1.1	4.6 ± 0.8
TG (mmol/L)	1.7 ± 0.1	1.8 ± 0.7^a^	2.1 ± 0.6^a,b^
HDL-C (mmol/L)	1.4 ± 0.3	1.2 ± 0.1	1.4 ± 0.3
LDL-C (mmol/L)	2.4 ± 0.9	3.2 ± 0.6^a^	3.9 ± 0.7^a,b^

^a^compared with non-CAD group

^b^compared with pre-menopausal CAD group

Among premenopausal women, MAPK-1 was indicated to be positively correlated with TG (rs = 0.271, P < 0.001), while HO-1 expressions increased with elevated CysC (*r*_s_ = 0.492, *P* < 0.001) and hs-CRP (*r*_s_ = 0.569, *P* < 0.001) (Table 5). Slightly different from premenopausal populations, postmenopausal ones revealed positive associations of MAPK-1 with CysC (*r*_s_ = 0.415, *P* < 0.001) and TG (*r*_s_ = 0.476, *P* < 0.001). The positive correlations between HIF-1 and either hs-CRP (*r*_s_ = 0.137, *P* = 0.005) or LDL-C (*r*_s_ = 0.470, *P* < 0.001) could also be found. Additionally, HO-1 exhibited positive linkages with CysC (*r*_s_ = 0.190, *P* < 0.001), hs-CRP (*r*_s_ = 0.542, *P* < 0.001) and TG (*r*_s_ = 0.142, *P* = 0.004) as well.

**Table 5:** Correlations between protein expressions (MAPK-1,HIF-1α and HO-1) and CAD-related indicators among groups of premenopausal and postmenopausal women

Indicators	Premenopausal-CAD group			Postmenopausal-CAD group		
	MAPK-1	HIF-1α	HO-1	MAPK-1	HIF-1α	HO-1
BMI (kg/m2)	−0.07 (0.39)	−0.02 (0.83)	0.05 (0.55)	−0.06 (0.23)	−0.02 (0.74)	−0.01 (0.84)
SBP (mm Hg)	−0.01 (0.93)	0.08 (0.32)	−0.09 (0.26)	0.05 (0.36)	−0.01 (0.83)	0.08 (0.12)
DBP (mm Hg)	0.11 (0.15)	0.02 (0.78)	0.09 (0.23)	−0.05 (0.3)	−0.02 (0.63)	0.07 (0.15)
Heart rate (/min)	−0.04 (0.59)	0.04 (0.65)	0.01 (0.91)	0.02 (0.66)	0.01 (0.88)	−0.06 (0.26)
FBG (mmol/L)	0.06 (0.41)	0.09 (0.23)	−0.1 (0.22)	0.01 (0.81)	0.04 (0.42)	0.04 (0.42)
2 h PG (mmol/L)	0.14 (0.08)	−0.06 (0.43)	−0.03 (0.7)	0.08 (0.1)	−0.01 (0.78)	0.02 (0.72)
BUN (mmol/L)	−0.13 (0.09)	0.04 (0.62)	0.06 (0.42)	−0.03 (0.59)	0.06 (0.24)	−0.01 (0.78)
Cr (μmol/L)	0.06 (0.43)	0.02 (0.78)	0.04 (0.65)	0.05 (0.35)	−0.01 (0.93)	0.00 (0.95)
CysC (mg/dL)	0.08 (0.31)	0.05 (0.52)	0.49 (<0.05)	0.42 (<0.05)	0.00 (0.98)	0.19 (<0.05)
hs-CRP (mg/L)	0.08 (0.29)	0.03 (0.75)	0.57 (<0.05)	0.08 (0.11)	0.14 (0.01)	0.54 (<0.05)
TC (mmol/L)	−0.15 (0.05)	0.08 (0.31)	0.01 (0.88)	0.01 (0.91)	0.00 (0.99)	0.06 (0.24)
TG (mmol/L)	0.27 (<0.05)	0.06 (0.43)	0.03 (0.72)	0.48 (<0.05)	0.04 (0.45)	0.14 (<0.05)
HDL-C (mmol/L)	0.01 (0.87)	0.07 (0.39)	−0.12 (0.13)	−0.02 (0.72)	0.01 (0.82)	0.03 (0.53)
LDL-C (mmol/L)	−0.15 (0.05)	0.08 (0.32)	−0.08 (0.33)	0.01 (0.92)	0.47 (<0.05)	0.01 (0.88)

^*^The results were presented as rs and *P* value

### Correlation between protein expressions relevant to MAPK signaling pathway and coronary angiography indicators

Interestingly, despite groups of non-CAD, pre-menopausal CAD and post-menopausal CAD, expressions of HIF-1α and HO-1 increased significantly with changes of lesion vessels (left main coronary artery < anterior descending artery < circumflex coronary artery < right coronary artery), number of stenosed coronary vessel (single vessel lesion < double vessel lesion < triple vessel lesion) and lesion type (type A < type B < type C)(*P* < 0.05) (Table 6). However, p38MAPK expressions stayed stable irrespective of the place of lesion vessels (*P* < 0.05), though its expressions also elevated with rise in number of stenosed coronary vessel and lesion type (*P* < 0.05).

**Table 6:** Correlations between protein expressions (MAPK-1,HIF-1αand HO-1) and coronary angiography indicators among groups of premenopausal and postmenopausal women

Indicators	Premenopausal-CAD group			Postmenopausal-CAD group		
	MAPK-1	HIF-1α (ng/L)	HO-1 (ng/L)	MAPK-1	HIF-1α (ng/L)	HO-1 (ng/L)
Lesion vessels						
Left main coronary artery	4.4 ± 0.1	13.8 ± 4.3	230.5 ± 50.2	4.7 ± 0.2	15.8 ± 4.2	264.3 ± 89.8
Anterior descending artery	4.5 ± 0.1	17.2 ± 7.3^*^	374.4 ± 195.3^*^	4.7 ± 0.12	21.4 ± 10.1^*^	397.7 ± 159.4^*^
Circumflex coronary artery	4.5 ± 0.1	20.3 ± 11.3^@^	419.6 ± 210.1^@^	4.8 ± 0.1	29.9 ± 12.1^@^	482.8 ± 278.4^@^
Right coronary artery	4.6 ± 0.2	24.1 ± 14.2^&^	527.1 ± 273.1^&^	4.8 ± 0.1	35.9 ± 15.0^&^	598.1 ± 262.1^&^
Number of stenosed coronary vessel						
Single vessel lesion	4.5 ± 0.2	16.0 ± 3.5	283.6 ± 82.0	4.7 ± 0.2	16.1 ± 3.7	278.4 ± 91.7
Double vessel lesion	4.5 ± 0.1^a^	19.3 ± 9.9^a^	409.7 ± 175.3^a^	4.7 ± 0.1^a^	23.6 ± 10.1^a^	349.8 ± 183.2^a^
Triple vessel lesion	4.5 ± 0.2^b^	29.1 ± 13.2^b^	561.6 ± 280.6^b^	4.8 ± 0.1^b^	39.6 ± 13.2^b^	637.9 ± 257.6^b^
Lesion type						
Type A	4.4 ± 0.2	14.7 ± 2.9	236.9 ± 90.2	4.7 ± 0.1	18.7 ± 4.5	293.5 ± 85.4
Type B	4.5 ± 0.1^c^	17.6 ± 8.1^c^	418.7 ± 161.3^c^	4.7 ± 0.1^c^	25.1 ± 7.9^c^	375.8 ± 155.5^c^
Type C	4.6 ± 0.2^d^	30.7 ± 11.4^d^	520.6 ± 224.5^d^	4.8 ± 0.2^d^	34.7 ± 10.8^d^	614.5 ± 249.5^d^

^*^: compared with left main coronary artery; ^@^: compared with anterior descending artery; ^&^: compared with circumflex coronary artery; ^a^: compared with single vessel lesion; ^b^: compared with double vessel; ^c^: compared with type A; ^d^: compared with type B

### Correlation between CAD-relevant indicators and Genesis score

It was observed that merely SBP and TG appeared as independently hazardous factors for prediction of CAD prognosis, with ORs of 1.02 (95 % CI = 1.01–1.18, *P* < 0.05) and 1.82 (95 % CI = 1.01–3.33, *P* < 0.05), respectively (Table 7). Nevertheless, none of any other available associations were found between CAD-indicators and Genesis score among both premenopausal and postmenopausal populations (all *P* > 0.05).

**Table 7:** Correlations between CAD-related indicators and Genisin scores

Items	Premenopausal-CAD group		Postmenopausal-CAD group	
	OR (95 % CI)	*P*	OR (95 % CI)	*P*
Hypertension	0.63 (0.29, 1.35)	0.232	0.91 (0.61, 1.36)	0.64
Hyperlipoidemia	2.63 (0.91, 7.62)	0.075	0.70 (0.45, 1.08)	0.11
Diabetes	0.95 (0.35, 2.56)	0.915	0.84 (0.54, 1.30)	0.43
Family History	0.95 (0.44, 2.06)	0.905	1.39 (0.83, 2.34)	0.21
Smoking History	1.38 (0.60, 3.16)	0.444	1.29 (0.80, 2.06)	0.29
MAPK-1	0.13 (0.00, 42.64)	0.488	0.41 (0.05, 3.48)	0.41
HIF-1α	1.02 (0.96, 1.08)	0.586	0.99 (0.96, 1.02)	0.52
HO-1	1.00 (1.00, 1.00)	0.966	1.00 (1.00, 1.00)	0.54
SBP (mm Hg)	1.02 (1.01, 1.18)	0.043	1.00 (0.99, 1.01)	0.98
CysC (mg/dL)	1.66 (0.47, 5.90)	0.433	0.95 (0.33, 2.75)	0.92
hs-CRP (mg/L)	0.99 (0.93, 1.05)	0.620	1.00 (0.97, 1.04)	0.88
TG (mmol/L)	1.82 (1.01, 3.33)	0.047	1.13 (0.72, 1.78)	0.59
LDL-C (mmol/L)	1.24 (0.63, 2.45)	0.537	1.10 (0.80, 1.51)	0.56

## Discussion

Among the classical factors, hypertension was demonstrated to accelerate progression of atherosclerosis through inducing continuous or repeated mechanical injuries of vascular endothelial cell layers [17]. Diabetes was always accompanied by numerous metabolic disorders, and it can even counteract the protective effects of estrogen [18]. Moreover, the role of dyslipidemia, such as high triglyceride (TG) in the plasm, in CAD development also appeared to be crucial since that TG enabled patients’ blood to be in a hypercoagulative state and thereby elevated CAD risk [19]. C-reactive protein (CRP) stimulated endothelial cells of aorta to generate high levels of plasminogen activator inhibitor-1 (PAI-1), which induce injuries of endarterium and instability of plaques [20]. Increased heart rate (HR) reduced oxygen supply and raised oxygen consumption, which spurred production of arteriosclerosis and augmented incidence of thrombus [21].

Besides, the onset of CAD seemed to be accompanied with myocardial hypoxia [22], and inhibited p38MAPK has been documented to worsen cellular injury under exposure to hypoxia [23, 24]. In fact, p38MAPK could be deemed as a candidate biomarker for CHD, owing to the facts that it mediated development of cardiac failure and that cardiac failure existed as a most severe disorder of CHD [25]. It was also documented that suppression of tumorigenicity 2 (ST2) could bind interleukin-33 (IL-33) on inflammatory membranes, thereby restraining and facilitating development of cardiovascular disorders [26, 27]. The in vivo/in vitro experiments conducted by Sanda et al. also demonstrated that administration of IL-33 could serve to lower p38MAPK expressions and Jun N-terminal kinase phosphorylation aroused by angiotensin II, which caused cardiac dysfunctions through generation of certain reactive oxygen species (ROS) [28]. All in all, the effects of p38MAPK on cardiovascular functions appeared to be regulated by ST2/IL-33 signaling, though the inherent pathogenesis needed to be further identified.

In addition, p38MAPK also remarkably up-regulated HIF-1α expressions in the hypoxic myocardium [29, 30]. The elevated HIF-1α expressions in acute ischemic myocardial tissue facilitated increase of capillary density within the damaged myocardium areas [31], where micro-circulation was effectively constructed [32]. Accumulating evidence also confirmed that the hypoxic pre-conditioning (HPC) mechanism mobilized by activated HIF-1α not only eased arrhythmia after ischemia-reperfusion via improving intracellular ion disorder, but also ameliorated blood supply by way of releasing endothelium-derived relaxing factor (EDRF) [33, 34]. Moreover, restrained HIF-1α expressions could antagonize the formation of new blood cells by repressing VEGF expressions, accordingly alleviating the incidence of in-stent restenosis when inhibitors of HIF-1α (e.g. mTOR and paclitaxel) were painted on the bracket [35].

As an endogenous protective factor, HO-1 was subject to regulation of HIF-1α [36]. Under the stimulation of ischemia and hypoxia, HO-1 functioned to resist atherosclerosis, ischemia repercussion injury and myocardial hypertrophy mainly through catalysing degradation of haem into bilirubin, CO and ferroprotein [37, 38]. Bilirubin has been documented to be associated with less susceptibility to CHD for its crucial roles in inhibition of lipid oxidation, clearance of ohyradicals and suppression of complement response during inflammation development [39–41]. In addition, CO could potentially block the inflammatory process through depressing generation of inflammatory factors that were relevant to NO and cGMP, whereas ferroprotein prevented cells from damage with its anti-oxidative capability [38, 42, 43].

In addition, postmenopause made female CAD patients more susceptible to hazard factors of cardiovascular diseases owing to shortage of estrogen. Estrogen not merely regulated blood lipid levels and reduced the chance of atherosclerosis, but also ameliorated a series of vascular endothelial functions, which were specifically indicated as dilation of coronary vessels, increase of coronary flow and reduction of coronary artery spasm. Furthermore, estrogen also inhibited migration of monocytes to subendothelial, and thereby restrained their transformation to foam cells after swallowing lipids [44]. Estrogen was also advantageous in restraining proliferation of vascular smooth muscle cells and antagonizing aggregation of platelets. In spite of the above merits, estrogen could aggravate premenopausal CAD when diabetes was combined, which might be explained by that estrogen can stimulate RAGE expressions within endothelial cells and potentiate advanced glycation end products (AGE)-RAGE interactions, finally exacerbating vascular inflammation [45].

Intriguingly, estrogen was also suggested to modulate p38 MAPK activity and HO-1 expressions [46]. Particularly, administration of 17 beta-estradiol (E(2)) for trauma-hemorrhage patients would normalized p38 MAPK, yet not HO-1 expressions, whereas addition of p38MAPK inhibitor abolished the increase of HO-1 expressions due to trauma haemorrhage [46]. It was also indicated that MAPK may phosphorylate certain targets (e.g. p27) that resulted in anti-estrogen resistance [47]. For another, E2 imposed effects primarily through binding to the estrogen receptors (ERα and ERbelta) or interacting with elements related with estrogen response [48]. Lower ERα expressions were determined in HIF-1α positive breast cancers than in HIF-1α ones [49]. Simultaneously, down-regulated ERα and inhibited influence of anti-estrogen could also be observed in the oxygen-lacking state [50], suggesting the inter-correlation between hypoxia, ERα and HIF-1α.

## Conclusions

Thus, it was reasonable to consider that p38MAPK-1/HIF-1α/HO-1 were highly associated with coronary lesion characteristics and prognosis for peri-menopausal women, providing clues that p38MAPK-1/HIF-1α/HO-1 could act as biomarkers for diagnosis and prognosis of peri-menopausal women suffering from CAD.

## Abbreviations

A-FABP: Adipocyte fatty acid-binding protein
BSA: Bovine serum albumin
CAD: Coronary artery disease
CRP: C-reactive protein
EDRF: Endothelium-derived relaxing factor
FDG: Fasting blood-glucose
HDL-C: High-density lipoprotein cholesterol
HR: Heart rate
IOD: Integrated optical density
LDL-C: Low-density lipoprotein cholesterol
p38MAPK: p38-mitogen activated protein kinase
PAI-1: Plasminogen activator inhibitor-1
PBS: Phosphate buffer saline
RBP4: Retinol-binding protein-4

## References

[1] De S, Searles G, Haddad H (2002). The prevalence of cardiac risk factors in women 45 years of age or younger undergoing angiography for evaluation of undiagnosed chest pain. Can J Cardiol.

[2] Regitz-Zagrosek V (2003). Cardiovascular disease in postmenopausal women. Climacteric.

[3] Ford ES, Capewell S (2007). Coronary heart disease mortality among young adults in the U.S. from 1980 through 2002: concealed leveling of mortality rates. J Am Coll Cardiol.

[4] Graham I, Atar D, Borch-Johnsen K, Boysen G, Burell G, Cifkova R, Dallongeville J, De Backer G, Ebrahim S, Gjelsvik B (2007). European guidelines on cardiovascular disease prevention in clinical practice: executive summary. Fourth Joint Task Force of the European Society of Cardiology and other societies on cardiovascular disease prevention in clinical practice (constituted by representatives of nine societies and by invited experts). Eur J Cardiovasc Prev Rehabil.

[5] Yusuf S, Reddy S, Ounpuu S, Anand S (2001). Global burden of cardiovascular diseases: part I: general considerations, the epidemiologic transition, risk factors, and impact of urbanization. Circulation.

[6] He J, Gu D, Wu X, Reynolds K, Duan X, Yao C, Wang J, Chen CS, Chen J, Wildman RP (2005). Major causes of death among men and women in China. N Engl J Med.

[7] Bao Y, Lu Z, Zhou M, Li H, Wang Y, Gao M, Wei M, Jia W (2011). Serum levels of adipocyte fatty acid-binding protein are associated with the severity of coronary artery disease in Chinese women. PLoS One.

[8] Lambadiari V, Kadoglou NP, Stasinos V, Maratou E, Antoniadis A, Kolokathis F, Parissis J, Hatziagelaki E, Iliodromitis EK, Dimitriadis G (2014). Serum levels of retinol-binding protein-4 are associated with the presence and severity of coronary artery disease. Cardiovasc Diabetol.

[9] Chen SM, Li YG, Zhang HX, Zhang GH, Long JR, Tan CJ, Wang DM, Fang XY, Mai RQ (2008). Hypoxia-inducible factor-1alpha induces the coronary collaterals for coronary artery disease. Coron Artery Dis.

[10] Holweg CT, Balk AH, Snaathorst J, van den Engel S, Niesters HG, Maat AW, Zondervan PE, Weimar W, Baan CC (2004). Intragraft heme oxygenase-1 and coronary artery disease after heart transplantation. Transpl Immunol.

[11] Yen ML, Su JL, Chien CL, Tseng KW, Yang CY, Chen WF, Chang CC, Kuo ML (2005). Diosgenin induces hypoxia-inducible factor-1 activation and angiogenesis through estrogen receptor-related phosphatidylinositol 3-kinase/Akt and p38 mitogen-activated protein kinase pathways in osteoblasts. Mol Pharmacol.

[12] Yeligar SM, Machida K, Kalra VK (2010). Ethanol-induced HO-1 and NQO1 are differentially regulated by HIF-1alpha and Nrf2 to attenuate inflammatory cytokine expression. J Biol Chem.

[13] Mao X, Wang T, Liu Y, Irwin MG, Ou JS, Liao XL, Gao X, Xu Y, Ng KF, Vanhoutte PM, Xia Z (2013). N-acetylcysteine and allopurinol confer synergy in attenuating myocardial ischemia injury via restoring HIF-1alpha/HO-1 signaling in diabetic rats. PLoS One.

[14] Nijm J, Jonasson L (2009). Inflammation and cortisol response in coronary artery disease. Ann Med.

[15] Wang M, Tsai BM, Reiger KM, Brown JW, Meldrum DR (2006). 17-beta-Estradiol decreases p38 MAPK-mediated myocardial inflammation and dysfunction following acute ischemia. J Mol Cell Cardiol.

[16] Evaluation EPOD. Executive Summary of The Third Report of The National Cholesterol Education Program (NCEP) Expert Panel on Detection, Evaluation, And Treatment of High Blood Cholesterol In Adults (Adult Treatment Panel III).[J]. JAMA. 2001;285(19):2486-2497. 10.1001/jama.285.19.2486 11368702

[17] Kannel WB (2000). Risk stratification in hypertension: new insights from the Framingham Study. Am J Hypertens.

[18] Kannel WB (1990). CHD risk factors: a Framingham study update. Hosp Pract (Off Ed).

[19] Carr MC, Brunzell JD (2004). Abdominal obesity and dyslipidemia in the metabolic syndrome: importance of type 2 diabetes and familial combined hyperlipidemia in coronary artery disease risk. J Clin Endocrinol Metab.

[20] Devaraj S, Xu DY, Jialal I (2003). C-reactive protein increases plasminogen activator inhibitor-1 expression and activity in human aortic endothelial cells: implications for the metabolic syndrome and atherothrombosis. Circulation.

[21] Giannoglou GD, Chatzizisis YS, Zamboulis C, Parcharidis GE, Mikhailidis DP, Louridas GE (2008). Elevated heart rate and atherosclerosis: an overview of the pathogenetic mechanisms. Int J Cardiol.

[22] Kyriakides ZS, Kremastinos DT, Michelakakis NA, Matsakas EP, Demovelis T, Toutouzas PK (1991). Coronary collateral circulation in coronary artery disease and systemic hypertension. Am J Cardiol.

[23] Sumbayev VV, Yasinska IM (2005). Regulation of MAP kinase-dependent apoptotic pathway: implication of reactive oxygen and nitrogen species. Arch Biochem Biophys.

[24] Yan HQ, Ma X, Chen X, Li Y, Shao L, Dixon CE (2007). Delayed increase of tyrosine hydroxylase expression in rat nigrostriatal system after traumatic brain injury. Brain Res.

[25] Pathan N, Franklin JL, Eleftherohorinou H, Wright VJ, Hemingway CA, Waddell SJ, Griffiths M, Dennis JL, Relman DA, Harding SE, Levin M (2011). Myocardial depressant effects of interleukin 6 in meningococcal sepsis are regulated by p38 mitogen-activated protein kinase. Crit Care Med.

[26] Kakkar R, Lee RT (2008). The IL-33/ST2 pathway: therapeutic target and novel biomarker. Nat Rev Drug Discov.

[27] Ciccone MM, Cortese F, Gesualdo M, Riccardi R, Di Nunzio D, Moncelli M, Iacoviello M, Scicchitano P (2013). A novel cardiac bio-marker: ST2: a review. Molecules.

[28] Sanada S, Hakuno D, Higgins LJ, Schreiter ER, McKenzie AN, Lee RT (2007). IL-33 and ST2 comprise a critical biomechanically induced and cardioprotective signaling system. J Clin Invest.

[29] Khandrika L, Lieberman R, Koul S, Kumar B, Maroni P, Chandhoke R, Meacham RB, Koul HK (2009). Hypoxia-associated p38 mitogen-activated protein kinase-mediated androgen receptor activation and increased HIF-1alpha levels contribute to emergence of an aggressive phenotype in prostate cancer. Oncogene.

[30] Caretti A, Morel S, Milano G, Fantacci M, Bianciardi P, Ronchi R, Vassalli G, von Segesser LK, Samaja M (2007). Heart HIF-1alpha and MAP kinases during hypoxia: are they associated in vivo?. Exp Biol Med (Maywood).

[31] Kido M, Du L, Sullivan CC, Li X, Deutsch R, Jamieson SW, Thistlethwaite PA (2005). Hypoxia-inducible factor 1-alpha reduces infarction and attenuates progression of cardiac dysfunction after myocardial infarction in the mouse. J Am Coll Cardiol.

[32] Su H, Kan YW (2007). Adeno-associated viral vector-delivered hypoxia-inducible gene expression in ischemic hearts. Methods Mol Biol.

[33] Cai Z, Zhong H, Bosch-Marce M, Fox-Talbot K, Wang L, Wei C, Trush MA, Semenza GL (2008). Complete loss of ischaemic preconditioning-induced cardioprotection in mice with partial deficiency of HIF-1 alpha. Cardiovasc Res.

[34] Weidemann A, Johnson RS (2008). Biology of HIF-1alpha. Cell Death Differ.

[35] Hong MK, Mintz GS, Lee CW, Song JM, Han KH, Kang DH, Song JK, Kim JJ, Weissman NJ, Fearnot NE (2003). Paclitaxel coating reduces in-stent intimal hyperplasia in human coronary arteries: a serial volumetric intravascular ultrasound analysis from the Asian Paclitaxel-Eluting Stent Clinical Trial (ASPECT). Circulation.

[36] Chung HT, Pae HO, Cha YN (2008). Role of heme oxygenase-1 in vascular disease. Curr Pharm Des.

[37] Song R, Kubo M, Morse D, Zhou Z, Zhang X, Dauber JH, Fabisiak J, Alber SM, Watkins SC, Zuckerbraun BS (2003). Carbon monoxide induces cytoprotection in rat orthotopic lung transplantation via anti-inflammatory and anti-apoptotic effects. Am J Pathol.

[38] Di Filippo C, Marfella R, Cuzzocrea S, Piegari E, Petronella P, Giugliano D, Rossi F, D’Amico M (2005). Hyperglycemia in streptozotocin-induced diabetic rat increases infarct size associated with low levels of myocardial HO-1 during ischemia/reperfusion. Diabetes.

[39] Novotny L, Vitek L (2003). Inverse relationship between serum bilirubin and atherosclerosis in men: a meta-analysis of published studies. Exp Biol Med (Maywood).

[40] Mayer M (2000). Association of serum bilirubin concentration with risk of coronary artery disease. Clin Chem.

[41] Djousse L, Levy D, Cupples LA, Evans JC, D’Agostino RB, Ellison RC (2001). Total serum bilirubin and risk of cardiovascular disease in the Framingham offspring study. Am J Cardiol.

[42] Peyton KJ, Reyna SV, Chapman GB, Ensenat D, Liu XM, Wang H, Schafer AI, Durante W (2002). Heme oxygenase-1-derived carbon monoxide is an autocrine inhibitor of vascular smooth muscle cell growth. Blood.

[43] Otterbein LE, Choi AM (2000). Heme oxygenase: colors of defense against cellular stress. Am J Physiol Lung Cell Mol Physiol.

[44] Chen L, Chester M, Kaski JC (1995). Clinical factors and angiographic features associated with premature coronary artery disease. Chest.

[45] Tanaka N, Yonekura H, Yamagishi S, Fujimori H, Yamamoto Y, Yamamoto H (2000). The receptor for advanced glycation end products is induced by the glycation products themselves and tumor necrosis factor-alpha through nuclear factor-kappa B, and by 17beta-estradiol through Sp-1 in human vascular endothelial cells. J Biol Chem.

[46] Hsu JT, Kan WH, Hsieh CH, Choudhry MA, Schwacha MG, Bland KI, Chaudry IH (2008). Mechanism of estrogen-mediated intestinal protection following trauma-hemorrhage: p38 MAPK-dependent upregulation of HO-1. Am J Physiol Regul Integr Comp Physiol.

[47] Donovan JC, Milic A, Slingerland JM (2001). Constitutive MEK/MAPK activation leads to p27(Kip1) deregulation and antiestrogen resistance in human breast cancer cells. J Biol Chem.

[48] Truss M, Beato M (1993). Steroid hormone receptors: interaction with deoxyribonucleic acid and transcription factors. Endocr Rev.

[49] Kurebayashi J, Otsuki T, Moriya T, Sonoo H (2001). Hypoxia reduces hormone responsiveness of human breast cancer cells. Jpn J Cancer Res.

[50] Kronblad A, Hedenfalk I, Nilsson E, Pahlman S, Landberg G (2005). ERK1/2 inhibition increases antiestrogen treatment efficacy by interfering with hypoxia-induced downregulation of ERalpha: a combination therapy potentially targeting hypoxic and dormant tumor cells. Oncogene.

